# Physiological profile of the Norwegian taekwondo (ITF) national team

**DOI:** 10.3389/fphys.2025.1661237

**Published:** 2025-11-11

**Authors:** Vegard V. Iversen, Arild B. Hafstad, Coral Falco, Morten Kristoffersen

**Affiliations:** Department of Sport, Food and Natural Sciences, Western Norway University of Applied Sciences, Campus Bergen, Bergen, Norway

**Keywords:** aerobic capacity, muscular strength, power, keiser, taekwondo, ITF

## Abstract

**Introduction:**

This study aimed to assess the physiological profile of the Norwegian National Taekwondo Team (ITF).

**Methods:**

Thirty-three athletes (Age = 20.8 ± 4.4), participated in the study, including 19 females (Age = 20.4 ± 3.4) and 14 males (Age = 21.6 ± 5.5). The athletes performed anthropometric and body composition, counter movement jump, maximal oxygen consumption (VO_2max_), leg press, brutal bench, and pull-ups tests. A Univariate Analysis of Variance (ANCOVA) was conducted to examine the effects on gender (male, female), experience (junior, senior), and competition level (medalists, non-medalists) on the outcome variables.

**Results:**

Athletes demonstrated the following anthropometric and physical characteristics: weight (males: 72.7 ± 11.7 kg; females: 61.7 ± 10.5 kg), height (males: 179.2 ± 5.3 cm; females: 168.9 ± 8.5 cm), body fat (males: 9.5% ± 4.4%; females: 18.6% ± 4.9%), VO_2_max (males: 58.8 ± 7.3 mL/kg/min; females: 50.5 ± 4.1 mL/kg/min) brutal bench (males: 15.6 ± 5.7 reps; females: 14.9 ± 8.9 reps), pull-ups (males: 11.4 ± 5.0 reps; females: 13.0 ± 9.3 reps), CMJ (males: 42.1 ± 4.8 cm; females: 31.1 ± 3.3 cm), maximum load (males: 234.0 ± 54.2 kg; females: 168.5 ± 33.6 kg), peak power (males: 1475.6 ± 390.0 W; females: 918.4 ± 162.4 W), and normalized peak power (males: 20.1 ± 2.8 W/kg females: 15.0 ± 1.6 W/kg).

**Conclusion:**

While physiological parameters show differences based on gender, they do not discriminate for competition level neither experience. Nevertheless, the Norwegian national team exhibit levels of physical and physiological conditions aligned with other elite athletes in other combat sports. The results provide valuable insights into the physiological levels that elite ITF taekwondo athletes should attain.

## Introduction

Taekwondo athletes require a wide range of physiological factors such as speed, strength, power, and endurance (Marković et al., 2005) and it has been suggested that no single physical attribute dominates performance (Beekley et al., 2006). Understanding the physiological characteristics of elite taekwondo athletes is crucial for optimizing performance and enhancing training methodologies (Zar et al., 2008; Zhu et al., 2025). International taekwondo athletes, generally, possess low levels of body fat, and while the VO_2max_ of taekwondo athletes is somewhat variable, it seems that moderate to high levels of anaerobic of physical fitness are necessary (Bridge et al., 2014; Franchini, 2023; Heller et al., 1998). However, there is evidence supporting the idea that taekwondo athletes should demonstrate superior performance in vertical jump height, higher upper and lower body strength, or superior anaerobic fitness to achieve success in international competition (Norjali et al., 2019; Sadowski et al., 2012) while research also supports the idea that muscle strength is often not a key role (Marković et al., 2005; Zhu et al., 2025). Taekwondo has two international governing bodies and while WT taekwondo has an extensive number of scientific studies (Millet et al., 2021), research on ITF is scarce (Poliszczuk et al., 2015; Góra et al., 2024). Moreover, as far as we know, only one study has analysed the level of physical fitness of ITF athletes (Heller et al., 1998). Providing objective feedback can enhance athlete motivation, particularly when it is grounded in a clear understanding of the performance standards required at various competitive levels. In recent years, the Norwegian national team has achieved notable success on the international stage, ranking 1st overall at the European ITF Taekwondo Championships and 6th overall at the World Championships. Despite this, there is a paucity of research that examines the physical attributes that contribute to their high-level performance. With this background in mind, the objective of the present research is to assess the physiological profile of the Norwegian National Taekwondo Team. In addition, we also aimed to analyse athletes based on competition level, experience, and gender.

## Methods

### Participants

Thirty-three taekwondo athletes (M_age_ = 20.8 ± 4), members of the Norwegian National Team, participated in the study. 19 were female (M_age_ = 20.4 ± 3.4) and 14 males (M_age_ = 21.6 ± 5.5). Regarding their experience, 10 athletes were juniors (M_age_ = 16.8 ± 0.4 and 23 seniors (M_age_ = 22.6 ± 4.1). Athletes who obtained a medal in an international competition, in the following European or World Championships were categorized as medallists. The study was approved by The Norwegian Data Protection Authority (Ref.#135031).

### Procedures

All tests were performed on the same day, in the same laboratory, under similar environmental conditions (18 °C–20 °C), The physical tests were performed with a minimum of 30 min breaks in between. The participants were instructed to have no strenuous exercise the day before testing and told not to eat and consume caffeine and nicotine products 3 h prior the tests. and in the following order.

### Anthropometry and body composition

Height was measured with a stadiometer (Seca 206 and Seca 217, Hamburg, Germany), recorded to the nearest cm. All measurements were performed barefoot using standard procedures. Body composition and body mass were estimated using an eight-polar bioimpedance method using a multifrequency current (InBody™ 720, Biospace CO., Ltd., Seoul, Korea).

### Brutal bench

The “brutal bench” is a vertical abdominal test similar to a sit-up (Gym 2000, Vikersund, Norway), but with higher demands for the hip flexors. Participants began in a vertical position with their feet secured at a 90-degree angle in the knee joint in the apparatus, their heads closest to the floor, and their hands positioned behind their heads. To ensure consistent hand placement throughout all repetitions, participants held onto a short, circular piece of rope. For each repetition, the elbows had to touch the knees. The maximum number of accepted repetitions was recorded and used for statistical analysis.

### Pull-up

The pull-up test was used to measure upper body strength. Male participants began in a vertical position with their arms fully extended while holding onto a bar. Female participants started in a horizontal position, with their bodies straight and supported by their heels on the floor. For each repetition, the chin had to rise above the bar. The maximum number of accepted repetitions was used for statistical analysis.

### Counter movement jump

The CMJ was conducted using both legs on a three-dimensional force plate (Kistler 9286B, Kistler Instruments AG, Winterthur, Switzerland). Participants began the CMJ from an upright stance and were instructed to descend to a self-selected depth before jumping vertically with maximum effort, with their hands on their hips. Each participant performed three jumps, and the best attempt was used for the statistical analysis.

### Leg strength and power

Maximal leg strength and power was assessed using a Keiser pneumatic leg press machine (Keiser A300, Keiser Co. Inc. United States). The corresponding Keiser A420 software (version 9.3.42) computes power as ( P=F·v ) calculated from pneumatic cylinder pressure and velocity (m/s) from displacement sensors. Participants were positioned in the machine with a knee angle of approximately 90°, and with their hands on the handles located on each side of the leg press seat. Two familiarization repetitions were followed by an estimation of 1RM and a 10-step protocol with increasing loads (Lindberg et al., 2021). Participants were also instructed to exert maximal effort on each repetition. A retrospective analysis of the tests confirmed that the 1RM estimations were justifiable, with participants averaging 10.6 ± 2.0 repetitions before reaching 1RM. Maximal load (kg), peak power (W), and normalized (by body weight) peak power (W/kg) were subsequently used in the statistical analysis.

### Aerobic capacity

Prior to testing, participants engaged in a 10-min warm-up by running on a treadmill (Woodway PPS55, United States) at a speed of 10 km/h. Maximal oxygen consumption (VO_2max_) was then measured using a standard protocol, constant incline of 5.3%, with the running velocity increased by 1 km/h every minute until the participant reached voluntary exhaustion. Gas exchange values were assessed using an Oxycon Pro apparatus (Jaeger GmbH, Hochberg, Germany) with a mixing chamber (Hans Rudolph Inc., Kansas City, MO, United States). Prior to each measurement, the oxygen (VO_2_) and carbon dioxide (VCO_2_) gas analysers were calibrated with high-precision gases (16.00% ± 0.04% O_2_ and 5.00% ± 0.1% CO_2_, Riesner-Gase GmbH and co, Lichtenfels, Germany). VO_2max_ was defined as the highest 1-min average VO_2_ during the test. During the tests, VO_2_ consumption, respiratory exchange ratio (RER), volume of expired air (VE), and HR were recorded and stored.

### Statistical analysis

Data normality for all performance variables (Weight, Height, BMI, Fat, VO_2max_, Brutal Bench, Pull-up, CMJ, Load, Peak Power, and Normalized Peak Power) were initially assessed using the Kolmogorov-Smirnov test. All variables, besides Peak Power (W) and Normalized Peak Power (W/kg), showed a normal distribution (p < 0.05). For the normal distributed variables, a Univariate Analysis of Variance (ANCOVA) was conducted to examine the effects of gender (male, female), experience (junior, senior), and competition level (medallists, non-medallists) followed by Tukey H pair subsequent comparisons. Partial eta-squared (η_p_^2^) or epsilon squared (ε^2^) values below 0.01, 0.01–0.06, 0.06–0.14, and above 0.14 were considered to have trivial, small, medium, and large effect sizes, respectively (Cohen, 1988). Moreover, it was analysed the 97% confidence interval and the Cohen’s d (effect sizes were considered as small [d = 0.2], medium [d = 0.5], and large [d = 0.8], respectively) (Cohen, 1988). For the non-normal distributed variables, an independent t-test was performed included ε^2^ to analyse the effect size. Statistical analyses were performed using Jamovi project (2024).

## Results

**FIGURE 1:**
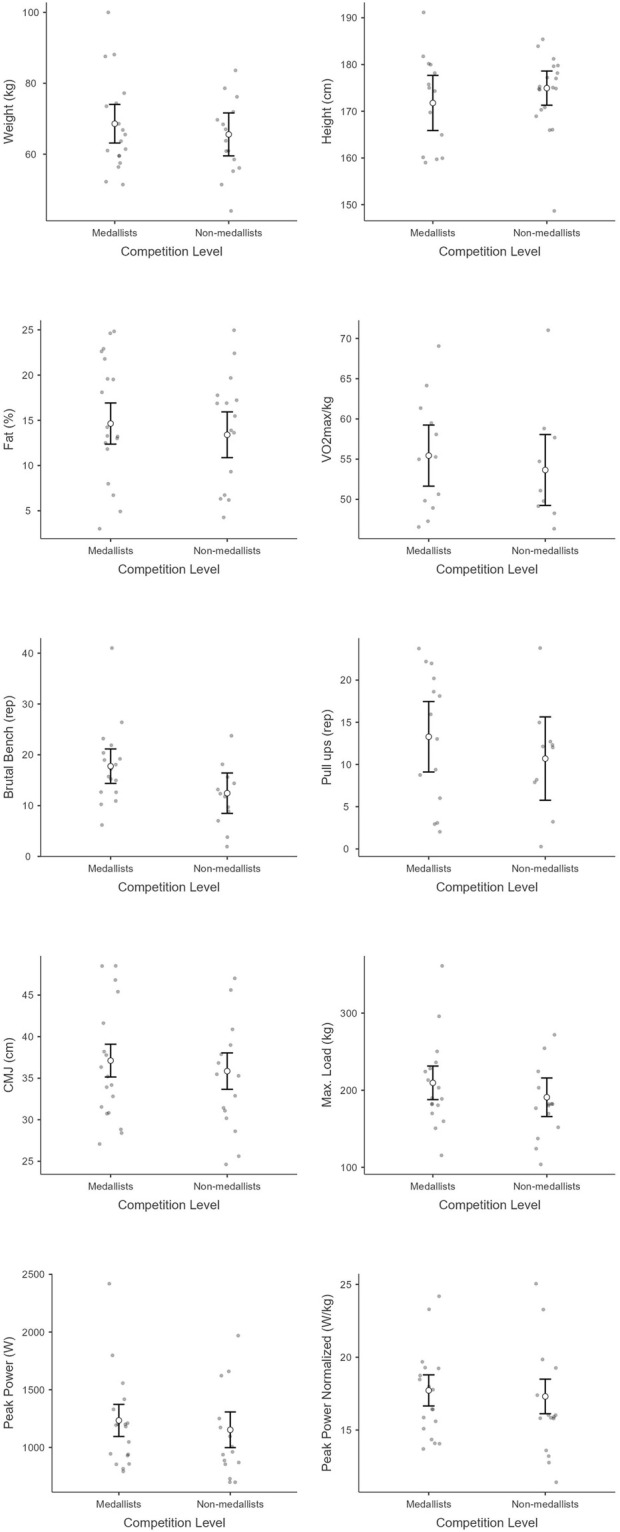
Physical and performance metrics between medalists and non-medalists regarding weight, height, body fat, VO_2max_/kg, brutal bench reps reps, pull-up reps, countermovement jump (CMJ) height, max load, peak power, and normalized peak power. Each plot shows individual data points, means and error bars to indicate variability within each level group.

**FIGURE 2:**
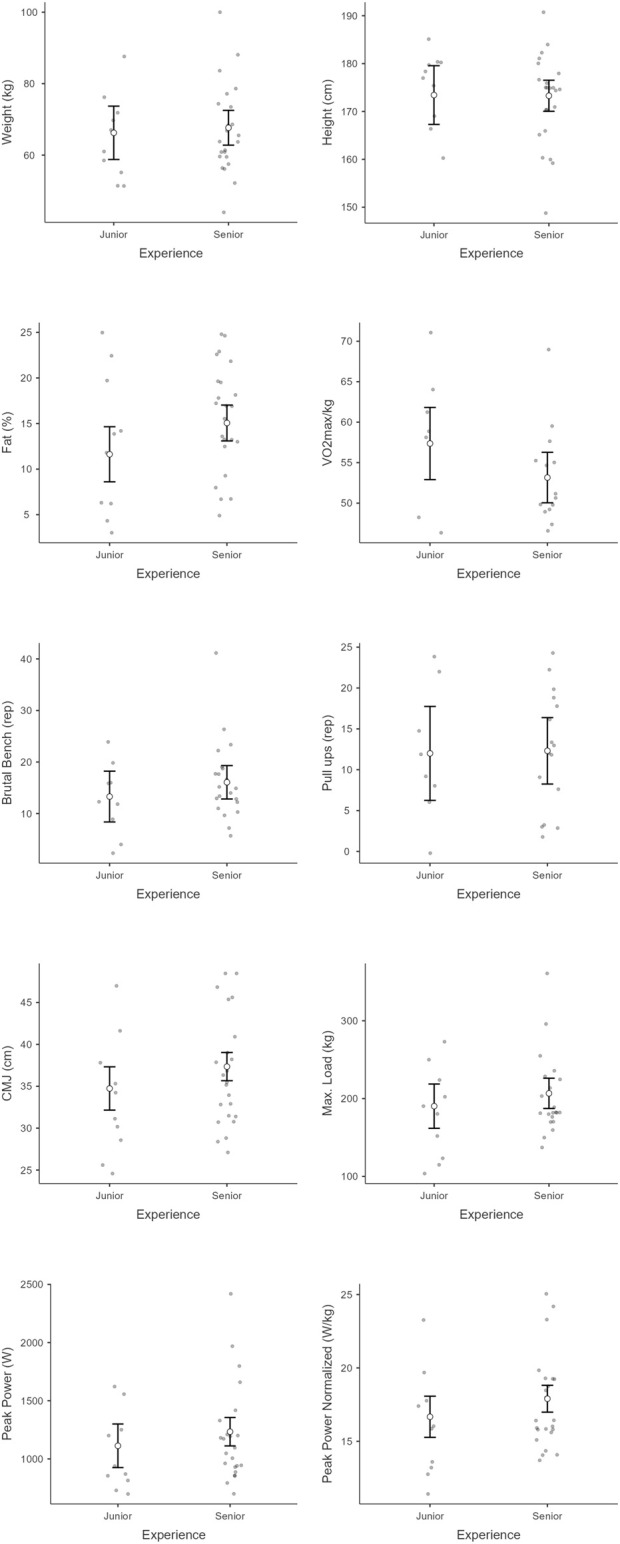
Physical and performance metrics comparing junior and senior athletes across eight measures: weight, height, body fat, VO_2max_/kg, brutal bench reps, pull-up reps, counter-movement jump (CMJ) height, max load, peak power, and normalized peak power. Each plot shows individual data points, means, and error bars to indicate variability within each experience group.

**FIGURE 3:**
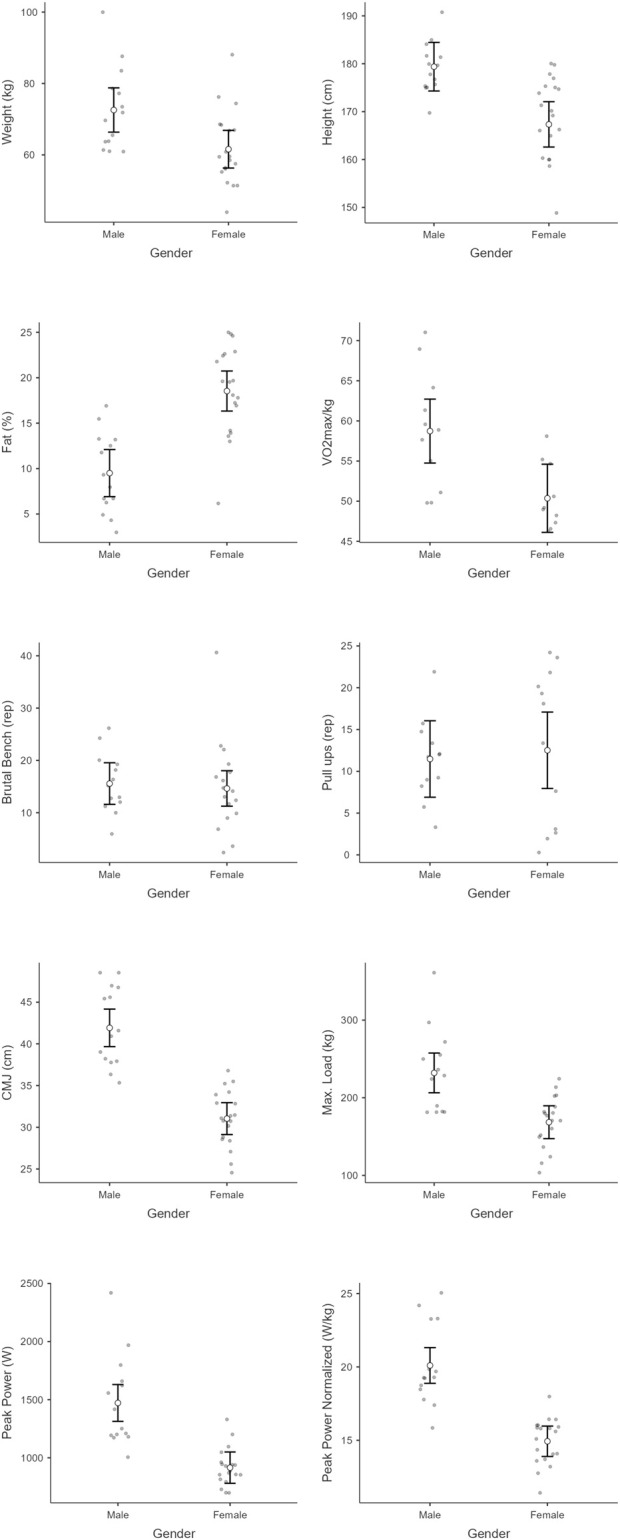
Physical and performance metrics comparing males and females across different metrics: weight, height, fat percentage, VO_2max_/kg, brutal bench reps, pull-up reps, counter-movement jump (CMJ) height, max load, peak power, and normalized peak power. Each plot shows individual data points, means, and error bars to indicate variability within each gender group.

Mean and standard deviation of each variable in each group and subgroup can be seen in Table 1 and Figure 1 (for competition level), Figure 2 (for experience), and Figure 3 (for gender), while Partial eta-squared (ηp^2^), confidence intervals (95% CI), and Cohen’s d are presented in Table 2.

**TABLE 1:** Descriptive statistics (mean and standard deviation) of the taekwondo athletes, according to their competition level (medallists = 18; non-medallists = 15), experience (junior = 10; senior = 23), and gender (male = 14; female = 19).

Variables	Gender	Medalists	Non-medalists	Junior	Senior	Total
		M ± SD	M ± SD	M ± SD	M ± SD	M ± SD
Weight (kg)	Male	73.7 ± 14.0	71.4 ± 8.6	72.6 ± 11.1	72.8 ± 12.5	72.7 ± 11.7^a^
	Female	63.5 ± 11.3	59.7 ± 9.7	60.0 ± 9.9	62.5 ± 11.1	61.7 ± 10.5^a^
	Total	68.1 ± 13.3	64.4 ± 10.7	64.9 ± 11.7	66.9 ± 12.5	66.4 ± 12.2
Height (cm)	Male	179.6 ± 6.4	178.8 ± 4.1	179.5 ± 4.2	179.1 ± 5.9	179.2 ± 5.3^a^
	Female	164.7 ± 7.0	170.7 ± 8.8	172 ± 8.3	167.5 ± 8.5	168.9 ± 8.5^a^
	Total	172.1 ± 10.0	174.11 ± 8.1	175.0 ± 7.7	172.5 ± 9.4	173.3 ± 8.9
Fat (%)	Male	9.2 ± 4.1	9.8 ± 5.2	6.4 ± 3.9	10.7 ± 4.1	9.5 ± 4.4^a^
	Female	20.1 ± 4.1	16.97 ± 5.5	16.9 ± 6.9	19.4 ± 3.8	18.6 + 4.9^a^
	Total	15.3 ± 6.8	14.1 ± 6.3	12.7 ± 7.8	15.6 ± 5.9	14.7 ± 6.5
VO_2max_ (mL/kg/min)	Male	59.8 ± 6.8	57.7 ± 8.5	63.8 ± 5.3	56 ± 6.9	58.8 ± 7.3^a^
	Female	51.1 ± 4.6	49.6 ± 3.6	50.9 ± 6.3	50.3 ± 3.4	50.5 ± 4.1^a^
	Total	55.4 ± 7.1	54.1 ± 7.7	58.3 ± 8.7	53.2 ± 5.9	54.9 ± 7.2
Brutal Bench (rep)	Male	15.8 ± 6.1	15.4 ± 5.6	18.0 ± 5.2	14.6 ± 5.9	15.6 ± 5.7
	Female	19.8 ± 8.96	9.5 ± 4.9	8.6 ± 5.7	17.6 ± 8.7	14.9 ± 8.9
	Total	17.9 ± 7.8	11.8 ± 5.8	12.8 ± 7.1	16.3 ± 7.6	15.2 ± 7.5
Pull ups (rep)	Male	11.1 ± 6.4	11.8 ± 2.5	10.5 ± 3.9	11.9 ± 5.6	11.4 ± 5.0
	Female	15.4 ± 9.1	9.6 ± 9.5	13.5 ± 11.5	12.8 ± 8.9	13.0 ± 9.3
	Total	13.3 ± 7.9	10.7 ± 6.6	12.0 ± 8.1	12.3 ± 7.2	12.2 ± 7.3
CMJ (cm)	Male	42.9 ± 5.0	40.95 ± 4.5	40.4 ± 5.1	42.7 ± 4.7	42.1 ± 4.8^a^
	Female	31.3 ± 2.7	30.7 ± 4.1	29.1 ± 3.6	31.9 ± 2.9	31.1 ± 3.3^a^
	Total	36.5 ± 7.1	34.8 ± 6.6	33.6 ± 7.1	36.7 ± 6.6	35.7 ± 6.8
Load_Max_ (kg)	Male	240,9 ± 62.5	223.1 ± 41.3	234.3 ± 35.4	233.9 ± 62.7	234.0 ± 54.2^a^
	Female	178.3 ± 33.9	158.7 ± 31.9	146.3 ± 39.2	179.6 ± 25.3	168.5 ± 33.6^a^
	Total	207.8 ± 57.7	181.7 ± 46.7	181.5 ± 57.8	202.9 ± 51.8	195.9 ± 53.8
Peak Power (W)	Male	1497.3 ± 432.9	1446.7 ± 362.2	1407.8 ± 212.5	1964.9 ± 169.6	1475.6 ± 390.0^a^
	Female	971.3 ± 174.4	859.6 ± 133.10	817.7 ± 90.1	964.9 ± 169.6	918.4 ± 162.4^a^
	Total	1205.1 ± 406.9	1094.4 ± 381.6	1053.7 ± 335.3	1198.7 ± 415.4	1154.8 ± 393.5
Peak Power_Norm_ (W/kg)	Male	20.1 ± 2.3	20.1 ± 3.49	19.5 ± 2.7	20.3 ± 2.9	20.1 ± 2.8^a^
	Female	15.4 ± 1.4	14.5 ± 1.8	13.8 ± 1.8	15.5 ± 1.2	15.0 ± 1.6^a^
	Total	17.46 ± 3.1	16.8 ± 3.8	16.1 ± 3.6	17.6 ± 3.2	17.1 ± 3.3

^a^ means significant differences between males and females.

Note: All tests were performed by 33 athletes, except for Brutal Bench (n = 30), Pull-ups (n = 24), and VO_2_max (n = 21).

**TABLE 2:** 95% Confidence intervals (95% CI), partial eta-square (ηp^2^) and Cohen’s d (d) according to Competition level (medallists and non-medallists), Experience (junior and senior) and Gender (male and female) of the analysed variables.

Variables	Gender			Competition level			Experience		
	95% CI	ηp^2^	d	95% CI	ηp^2^	d	95% CI	ηp^2^	d
Weight (kg)	[0.22–2.13]	0.23	1.17	[-0.84–0.94]	0	0.05	[-1.9–0.69]	0	−0.2
Height (cm)	[-2.65 to −0.57]	0.34	−1.61	[-1.36–0.51]	0.03	−0.42	[-0.95–0.91]	0	−0.02
Fat (%)	[-2.90 to −0.82]	0.43	−1.86	[-0.84–0.94]	0	0.05	[-1.72–0.12]	0.12	−0.8
VO_2max_ (mL/kg/min)	[0.25–2.69]	0.41	1.47	[-0.53–1.62]	0.9	0.55	[-0.19–2.06]	0.22	0.94
Brutal Bench (rep)	[-0.63–0.91]	0	0.14	[-0.20–1.85]	0.1	0.83	[-1.04–0.91]	0	−0.06
Pull ups (rep)	[-1.34–0.67]	0.03	−0.34	[-0.58–1.44]	0.05	0.43	[-0.75–1.25]	0.02	0.25
CMJ (cm)	[1.37–3.67]	0.58	2.52	[-0.58–1.20]	0.02	0.31	[-1.35–0.44]	0.04	−0.45
LoadMax (kg)	[0.69–2.79]	0.41	1.74	[-0.93–0.88]	0	−0.02	[-1.32–0.51]	0.04	−0.4

Regarding weight, there was a significant main effect of gender [F_(1,25)_ = 7.4, p =0 .01]. However, there was a non-significant main effect of competition level [F_(1, 25)_ = 0.1, p = 0.92,] nor experience [F_(1, 25)_ = 0.20, p = 0.65]. The interaction effects were therefore non-significant (p > 0.05). For height, there was a significant main effect of gender [F_(1, 25)_ = 12.78, p < 0.001]. However, there was a non-significant main effect of competition level [F_(1, 25)_ = 0.89, p = 0.36] nor experience [F_(1, 25)_ = 0.01, p = 0.97]. The interaction effects were therefore non-significant (p > 0.05). A significant main effect of gender was observed for body fat [F _(1, 25)_ = 18.65, p < 0.001]. However, there was not a significant main effect of competition level [F_(1, 25)_ = 0.1, p = 0.91] nor experience [F_(1, 25)_ = 3.43, p = 0.08]. The interaction effects were therefore non-significant (p > 0.05). In terms of VO_2max_, there was a significant main effect of gender [F_(1, 13)_ = 9.2, p = 0.01]. However, there was not a significant main effect of competition level [F_(1, 13)_ = 1.26, p = 0.28] nor experience [F_(1, 13)_ = 3.72, p = 0.08]. The interaction effects were therefore non-significant (p > 0.05). Regarding brutal bench, there was a non-significant main effect of gender [F_(1, 25)_ = 0.14, p = 0.72], competition level [F_(1, 25)_ = 4.4, p = 0.05] nor experience [F_(1, 25)_ = 0.93, p = 0.34]. The interaction effects were therefore non-significant (p > 0.05). For Pull-ups there was a non-significant main effect of gender [F_(1, 20)_ = 0.51, p = 0.48], competition level [F_(1, 20)_ = 0.83, p = 0.38] nor experience [F_(1, 20)_ = 0.29, p = 0.60]. The interaction effects were therefore non-significant (p > 0.05). In CMJ, there was a significant main effect of gender [F_(1, 25)_ = 34.12, p < 0.001]. However, the main effect of competition level [F_(1, 25)_ = 0.52, p = 0.48] nor experience [F_(1, 29)_ = 1.11, p = 0.30] was non-significant. The interaction effects were therefore non-significant (p > 0.05). Regarding load, there was a significant main effect of gender [F_(1, 23)_ = 15.74, p < 0.001]. However, the main effect of competition level [F_(1, 23)_ = 0.01, p = 0.96] was not significant nor the main effect of experience [F_(1, 23)_ = 0.85, p = 0.37]. The interaction effects were therefore non-significant (p > 0.05).

For peak power, there were significant differences in gender (χ^2^ = 18.95, p < 0.001, ε^2^ = 0.59). However, there were no significant differences regarding competition level (χ^2^ = 0.95, p = 0.33, ε^2^ = 0.03) neither regarding experience (χ^2^ = 1.25, p = 0.26, ε^2^ = 0.04). Regarding normalized peak power, there were significant differences in gender (χ^2^ = 20.40, p < 0.001, ε^2^ = 0.64) no significant differences regarding competition level (χ^2^ = 0.69, p = 0.41, ε^2^ = 0.02) neither regarding experience (χ^2^ = 1.38, p = 0.24, ε^2^ = 0.04).

## Discussion

This study aimed to assess the physiological profile of the Norwegian National Taekwondo (ITF) Team. In addition, we also aimed to analyse athletes based on competition level, experience, and gender.

In terms of body fat, females showed a higher percentage than males (high effect size). Our results are similar to previous studies (Bridge et al., 2014; Reale et al., 2020) and lower than those reported by Kim and Nam (2021) from Korean elite taekwondo athletes. No differences were found regarding competition level nor experience.

Regarding VO_2max_, and in line with previous studies (Mathunjwa et al., 2015; Kim and Nam, 2021; Heller et al., 1998), males showed higher values than females (high effect size). However, no differences were found regarding competition level nor experience (moderate effect size). It is interesting to highlight that our results are higher than those reported by Franchini et al. (2011) in elite judo, by Chabene et al. (2012) in karate athletes, or by Heller et al. (1998) for the ITF Czech national team, and slightly higher than those reported by Mathunjwa et al. (2017) or Kim and Nam (2021) for male and female senior taekwondo athletes. In any case, it seems that aerobic capacity is essential in combat sports (Zhang et al., 2025; Kirk et al., 2024; Ruddock et al., 2021). Our results indicates that the Norwegian team is well inside the international aerobic levels, as well as showing that the level of preparation of the athletes has increased over the years. The results also show that Taekwondo ITF is an aerobically demanding sport.

To our knowledge, no previous studies in combat sports have utilized the brutal bench and the pull-up tests, since most of the studies have used 60-s sit-up tests in combat sports athletes (Marković et al., 2005; Noorul et al., 2008; Sadowski et al., 2012; Toskovic et al., 2004). Therefore, the direct comparisons is challenging. Moreover, the effect size was moderate to large, suggesting that despite non-significance, the observed difference may reflect limited statistical power. However, the brutal bench is considered relevant for taekwondo athletes as it engages the hip flexors and abdominal muscles, which are important in this sport (Jeon et al., 2021). Our results are in line with previous studies that found no differences between two different levels of taekwondo athletes (Baldi et al., 1990; Toskovic et al., 2004), neither regarding gender (Noorul et al., 2008) and in contrast to those that has reported significant differences based on competition level (Marković et al., 2005; Sadowski et al., 2012), and gender (Toskovic et al., 2004). The lack of significant differences in the Norwegian team could be explained by the fact that all athletes are members of the national team, and therefore, all of them are at a high level of performance.

In line with previous studies (Noorul et al., 2008; Marković et al., 2005), no differences were found regarding competition level and gender in pull-ups (small to moderate effect size). Our results are higher than the ones reported by Fajar et al. (2021) and extend previous findings suggesting that endurance properties of the upper extremities may be important to support several technical and tactical actions in combat, but it does not determine success in international competition. Nevertheless, the available data will provide coaches and trainers insight into the muscular endurance characteristics of ITF taekwondo athletes.

When it comes to the countermovement jump, and in line with previous studies in combat sports (Batra et al., 2025; Bridge et al., 2014; Chabene et al., 2012; Noorul et al., 2008; Škugor et al., 2023), males jumped higher than female athletes. However, no differences were found (small effect size) regarding competition level nor experience. Škugor et al. (2023) reported a significantly higher jump height for male national wrestler medallists compared to non-medallists, thus describing a difference in competition level not found in our study. Furthermore, Norjali et al. (2019) found significant differences between elite and non-elite collegiate taekwondo male athletes. Performance in combat sports largely depends on the ability to generate high power, which involves producing significant kinetic energy in a short time (Ravier et al., 2004; Blazević et al., 2006). However, it seems that when analysing high level athletes, it is difficult to determine one specific variable that discriminates the best from the second best. In any case, the findings of the present study confirms that male and female athletes performed within the range of reported averages for CMJ performance.

In general, maximal leg strength values were similar to the ones reported by Toskovic et al. (2004) for taekwondo athletes. In contrast, Sbriccoli et al. (2007) reported maximal leg press strength for Olympic Judo athletes considerably higher than what the athletes in the current study achieved. However, the Judo athletes had on average a much higher body weight in addition to lower VO_2max_ values than the athletes in the current study, which highlights the distinct difference in physiological demands in these two combat sports. Furthermore, Sbriccoli et al. (2007) did not include the protocol for measuring maximal leg strength which make maximal strength comparison difficult.

Regarding peak power (W), no differences were found according to the competition level neither experience. However, differences emerged according to gender. It can be noted that elite Norwegian Taekwondo athletes showed lower values compared to their conte-partner athletes from the same country, in non-comparable sports like ice hockey, handball, and alpine skiing (Nysether et al., 2023). Previous studies on combat sports have used the Wingate test or short duration cycle sprints to assess anaerobic power (Bridge et al., 2014; Chabene et al., 2012; Venckunas et al., 2024). To our knowledge, this is the first study utilizing the Keiser leg press to assess anaerobic power in combat athletes. It can be argued that assessing peak power via a leg press is more relevant for explosive movements such as jumping and kicking, as opposed to the Wingate cycle test, which determine peak power over a 5 s period, and may be influenced by the ability to maintain power output and cycling technique. However, since Wingate is the gold standard for testing anaerobic power, more research should be done to assess which test is more valid in assessing power in combat athletes.

Normalized peak power values appear to be higher in the Keiser leg press compared to the Wingate test (unpublished data), which is expected given that the Keiser leg press measures peak power over a shorter time interval than the Wingate test. It is interesting to notice that our results are in line (similar W/kg) with those reported by Ravier et al. (2004) in male Karate athletes or by Sadowski et al. (2012) in taekwondo athletes (WT). In contrast to our results, the beforementioned studies found superior anaerobic power in international athletes compared to national athletes, and medallists compared to non-medallists, respectively.

It has been reported that the athlete with the best physical fitness has the greatest probability of winning a championship, especially for female taekwondo athletes (Liu and He, 2022). However, in line with Heller et al. (1998) and Marković et al. (2005), our results indicate that physiological parameters do not evidently discriminate by competition level neither experience. The lack of significance might be explained by the fact that the tested athletes are at the top level, and differences are based on very small details, but also by the limited statistical power (due to low number of participants) of the study. Actually, *post hoc* computed statistical power showed that only variables brutal bench for competition level and VO_2max_ for experience achieved a “decent” (≈63%) power. Therefore, future research should take into account the sample size before conducting the experiment, and also focus on technical, tactical and mental toughness evaluation of performance to find measures of success beyond physiological traits. Nevertheless, the results of the present study give actual and updated data to athletes and coaches that would like to face international competitions in ITF taekwondo with enough guaranties that the physiological needs are met.

## Conclusion

The Norwegian national team is generally comparable to athletes in other elite combat sports even though group-level physiological parameters do not discriminate between performance levels or experience and are not direct indicators of success in championships. Nevertheless, the results provide valuable insights into the physiological levels that elite taekwondo athletes should attain, particularly within the Norwegian national team, but also among elite athletes in the International Taekwon-Do Federation.

## Data Availability

The raw data supporting the conclusions of this article will be made available by the authors, without undue reservation.

## References

[B1] Baldi M. Dianno M. V. Andrade D. R. Pereira M. H. N. (1990). Comparison of physical fitness of two different levels of taekwondo athletes. Rev. Bras. Ciência Mov. 4, 26–31.

[B2] Batra A. Finlay M. Kirk C. (2025). Jump performance and field-based anaerobic capacity profiles of international standard amateur mixed martial arts athletes. Rev. Artes Marciales Asiáticas 20 (1), 18–29. 10.18002/rama.v20i1.2502

[B3] Beekley M. Abe T. Kondo M. Midorikawa T. Yamauchi T. (2006). Comparison of normalized maximum aerobic capacity and body composition of sumo wrestlers to athletes in combat and other sports. J. Sports Sci. Med. 5, 13–20. 24357971 PMC3863922

[B4] Blazević S. Katić R. Popović D. (2006). The effect of motor abilities on karate performance. Coll. Antropol. 30 (2), 327–333. 16848147

[B5] Bridge C. A. Ferreira da Silva Santos J. Chaabene H. Pieter W. Franchini E. (2014). Physical and physiological profiles of taekwondo athletes. Sports Med. 44 (6), 713–733. 10.1007/s40279-014-0159-9 24549477

[B6] Chabene H. Hachana Y. Franchini E. Mkaouer B. Chamari K. (2012). Physical and physiological profile of elite karate athletes. Sports Med. 42 (10), 829–843. 10.1007/BF03262297 22901041

[B7] Cohen J. (1988). Statistical power analysis for the behavioral sciences. New York, NY: Routledge Academic.

[B8] Fajar M. K. Marsudi I. Rasyid A. Pramono B. A. Fepriyanto A. (2021). “Profile of taekwondo athletes in situbondo Indonesian,” in Proceedings of the International Joint Conference on Arts and Humanities (IJCAH 2021) (Paris, France: Atlantis Press), 503–506. 10.2991/assehr.k.211223.087

[B9] Franchini E. (2023). Energy system contributions during olympic combat sports: a narrative review. Metabolites 13, 297. 10.3390/metabo13020297 36837916 PMC9961508

[B37] Franchini E. Del Vecchio F. B. Matsushigue K. A. Artioli G. G. (2011). Physiological profiles of elite judo athletes. Sports Med. 41 (2), 147–166. 10.2165/11538580-000000000-00000 21244106

[B10] Góra T. Mosler D. Langfort J. Wąsik J. (2024). Differences in impact force between side kicks and turning kicks in Male practitioners of taekwon-Do—Case studies. Appl. Sci. 14 (13), 5876. 10.3390/app14135876

[B11] Heller J. Peric T. Dlouhá R. Kohlíková E. Melichna J. Nováková H. (1998). Physiological profiles of male and female taekwon-do (ITF) black belts. J. Sports Sci. 16 (3), 243–249. 10.1080/026404198366768 9596358

[B31] Jamovi project (2024). Jamovi. Available online at: https://www.jamovi.org.

[B12] Jeon Y. Kim Y. Ji Y. (2021). Specific muscle coordination patterns of taekwondo roundhouse kick in athletes and non-athletes. Archives Budo 17, 135–144.

[B13] Kim J. W. Nam S. S. (2021). Physical characteristics and physical fitness profiles of Korean taekwondo athletes: a systematic review. Int. J. Environ. Res. Public Health 18 (18), 9624. 10.3390/ijerph18189624 34574549 PMC8466171

[B14] Kirk C. Clark D. Langan-Evans C. (2024). The influence of aerobic capacity on the loads and intensities of mixed martial arts sparring bouts. J. Sports Sci. 42 (19), 2093–2102. 10.1080/02640414.2024.2419239 39551930

[B15] Lindberg K. Solberg P. Bjørnsen T. Helland C. Rønnestad B. Thorsen F. M. (2021). Force-velocity profiling in athletes: reliability and agreement across methods. PLoS ONE 16 (2), e0245791. 10.1371/journal.pone.0245791 33524058 PMC7850492

[B16] Liu R. He L. (2022). The relationship between physical fitness and competitive performance of taekwondo athletes. PLoS ONE 17 (6), e0267711. 10.1371/journal.pone.0267711 35749558 PMC9232157

[B17] Marković G. Misigoj-Duraković M. Trninić S. (2005). Fitness profile of elite Croatian female taekwondo athletes. Coll. Antropol. 29 (1), 93–99. 16117305

[B18] Mathunjwa M. L. Mugandani S. C. Ngcobo M. Djarova-Daniels T. Ivanov S. (2015). Physical, anthropometric and physiological profiles of experienced junior male and female South African taekwondo athletes. Afr. J. Phys. Health Educ. Recreat. Dance 21 (4:2), 1402–1416.

[B19] Mathunjwa M. L. Mugandani S. Kappo A. Ivanov S. Djarova-Daniels T. (2017). Motor ability profile of junior and senior male South African taekwondo athletes. J. Appl. Sports Sci. 2, 3–9. 10.37393/jass.2017.02.1

[B20] Millet G. P. Brocherie F. Burtscher J. (2021). Olympic sports science—Bibliometric analysis of all summer and winter olympic sports research. Front. Sports Act. Living 3, 772140. 10.3389/fspor.2021.772140 34746779 PMC8564375

[B21] Noorul H. R. Pieter W. Erie Z. Z. (2008). Physical fitness of recreational adolescent taekwondo athletes. Braz. J. Biomotricity 2 (4), 230–240.

[B22] Norjali W. M. R. W. Van Hiel M. Mostaert M. Deconinck F. J. A. Pion J. Lenoir M. (2019). Identification of elite performance characteristics in a small sample of taekwondo athletes. PLoS ONE 14 (5), e0217358. 10.1371/journal.pone.0217358 31150424 PMC6544235

[B23] Nysether S. Hopkins W. G. Mentzoni F. Paulsen G. Haugen T. A. Solberg P. A. (2023). Monitoring changes in lower-limb strength and power in elite athletes with the countermovement-jump and keiser leg-press tests. Int. J. Sports Physiology Perform. 18 (10), 1145–1151. 10.1123/ijspp.2023-0088 37451684

[B24] Poliszczuk T. Jankowska E. Mańkowska M. Poliszczuk D. Omiecinska I. (2015). Profile of an ITF Taekwon-do female champion team in terms of somatotype and BodyComposition. Archives Budo 11, 173–185.

[B25] Ravier G. Grappe F. Rouillon J. D. (2004). Application of force-velocity cycle ergometer test and vertical jump tests in the functional assessment of karate competitor. J. Sports Med. Phys. Fit. 44 (4), 349–355. 15758845

[B26] Reale R. Burke L. M. Cox G. R. Slater G. (2020). Body composition of elite olympic combat sport athletes. Eur. J. Sport Sci. 20 (2), 147–156. 10.1080/17461391.2019.1616826 31092119

[B27] Ruddock A. James L. French D. Rogerson D. Driller M. Hembrough D. (2021). High-intensity conditioning for combat athletes: practical recommendations. Appl. Sci. 11 (22), 10658. 10.3390/app112210658

[B28] Sadowski J. Gierczuk D. Miller J. Cieśliński I. Buszta M. (2012). Success factors in male WTF taekwondo juniors. J. Combat Sports and Martial Arts 1, 47–51. 10.5604/20815735.1047647

[B29] Sbriccoli P. Bazzucchi I. Di Mario A. Marzattinocci G. Felici F. (2007). Assessment of maximal cardiorespiratory performance and muscle power in the Italian olympic judoka. J. Strength and Cond. Res. 21 (3), 738–744. 10.1519/R-20245.1 17685696

[B30] Škugor K. Gilić B. Karninčić H. Jokai M. Babszky G. Ranisavljev M. (2023). What determines the competitive success of young Croatian wrestlers: anthropometric indices, generic or specific fitness performance? J. Funct. Morphol. Kinesiol. 8 (3), 90. 10.3390/jfmk8030090 37489303 PMC10366759

[B32] Toskovic N. N. Blessing D. Williford H. N. (2004). Physiologic profile of recreational male and female novice and experienced tae kwon Do practitioners. J. Sports Med. and Physcal Fit. 44, 164–172. 15470314

[B33] Venckunas T. Bruzas V. Snieckus A. Stasiule L. Kniubaite A. Mickevicius M. (2024). Anaerobic performance profiling in elite amateur boxers. Sports 12 (9), 231. 10.3390/sports12090231 39330708 PMC11435942

[B34] Zar A. Gilani A. Ebrahim K. H. Gorbani M. H. (2008). A survey of the physical fitness of the male taekwondo athletes of the Iranian national team. Phys. Educ. Sport 6 (1), 21–29.

[B35] Zhang Y. Chen L. Wang J. Li H. Liu X. (2025). Effects of high-intensity interval training on aerobic and anaerobic capacity in olympic combat sports: a systematic review and meta-analysis. Front. Physiology 16, 1576676. 10.3389/fphys.2025.1576676 PMC12098572 40415789

[B36] Zhu Y. Li Y. Wenren Y. Gao P. (2025). Physical fitness parameters of Chinese elite taekwondo athletes. Int. J. Morphol. 43 (3), 809–815. 10.4067/S0717-95022025000300809

